# Prevalence of monoclonal gammopathy of undetermined significance in a large population with annual medical check-ups in China

**DOI:** 10.1038/s41408-020-0303-8

**Published:** 2020-03-09

**Authors:** Jian-hua Han, Ji-nuo Wang, Yue-lun Zhang, Xin-xin Cao, Dao-bin Zhou, Teng-da Xu, Wei Su, Jian Li

**Affiliations:** 1 Department of Clinical Laboratory, Peking Union Medical College Hospital, Chinese Academy of Medical Sciences and Peking Union Medical College, Beijing, China; 2 Department of Hematology, Peking Union Medical College Hospital, Chinese Academy of Medical Sciences and Peking Union Medical College, Beijing, China; 3 Medical Research Center, Peking Union Medical College Hospital, Chinese Academy of Medical Sciences and Peking Union Medical College, Beijing, China; 4 Department of Health Medicine, Peking Union Medical College Hospital, Chinese Academy of Medical Sciences and Peking Union Medical College, Beijing, China

**Keywords:** Epidemiology, Cancer epidemiology, Risk factors

Dear Editor,

Monoclonal gammopathy of undetermined significance (MGUS) is a clinically asymptomatic premalignant plasma cell disorder, which may develop to multiple myeloma (MM), light-chain amyloidosis, Waldenström macroglobulinemia, or other related malignancies^1^. The crude prevalence of MGUS varies from 0.05% to 6.1% among distinct population from different countries, which is influenced by race, age, sex, family history, immunosuppression, and pesticide exposure^2^. So far, many population-based studies of MGUS come from the white or black population living in Western and African countries. One of the largest population-based study in the USA showed that the prevalence of MGUS was 3.2% in the Caucasian general population aged ≥50 years^3^, while the rate was even higher in African-Americans to be 3.7%^4^. However, data on the epidemiology of MGUS remain largely undefined in Asians. Some population-based studies among population from Thailand, Korea, and Hong Kong were relatively limited due to small sample sizes^5–7^. Therefore, we conducted this study to determine the prevalence and characteristics of MGUS among a large population with annual medical check-ups in a well-defined geographic area in China.

From December 2013 to April 2019, a total of 316,616 people received annual medical check-ups at the Peking Union Medical College Hospital. Generally, people who received annual medical check-ups at our institute were apparently healthy and reflected a population with relatively high social economic status and health consciousness in Beijing, China. All the people with measurement of serum protein electrophoresis were included in our analysis. People were excluded if age or sex was missing in the record. The information collected at the first time when the person came to our institute was regarded as baseline data, while consecutive follow-up records of the same participant were also collected (Supplementary Fig. 1). This study was approved by the institutional ethics committee of Peking Union Medical College Hospital and informed consent was waived because there is no information that can help to identify individuals.

MGUS was defined in accordance with previous definition by International Myeloma Working Group^8^. Serum M protein was detected by capillary electrophoresis (Sebia, Parc Technologique Leonard de Vinci, Cedex, France). Patients with a positive or suspicious M protein were suggested to be referred to the hematological clinic for further assessment, including serum and/or urine immunofixation electrophoresis (IFE) to determine the type of immunoglobulin and serum free light chain (FLC) assays for quantification. Serum IFE was performed using the SPIFE^®^ ImmunoFix-9 Kit (Helena Laboratories, TX, USA). Urine IFE was detected by the HYDRAGEL 9 IF Kit (Sebia, Parc Technologique Leonard de Vinci, Cedex, France). FLC measurements were performed by an IMMAGE 800 automated nephelometer (Beckman Coulter, CA, USA) using commercially available kits (Hevylite^®^, The Binding Site Ltd, Birmingham, UK). Published reference ranges for κ and λ FLC were used (3.3–19.4 and 5.7–26.3 mg/L, respectively). An abnormal κ/λ FLC ratio was defined as <0.26 or >1.65^9^. Serum concentrations of immunoglobulin G (IgG), immunoglobulin M (IgM), and immunoglobulin A (IgA) were evaluated by serum immunoglobulin assay.

The overall prevalence of MGUS was calculated by the number of people with MGUS in each age and sex stratum divided by the number of subjects in that stratum who underwent a medical check-up. Descriptive statistics included medians with minimum and maximum for continuous variables and counts and percentages for categorical variables. Exact 95% confidence intervals (CIs) for prevalence were computed using the binomial distribution. Differences between groups normally distributed with homogeneity of variance were analyzed with Student’s *t* test. All tests conducted were two tailed, and *P* values <0.05 were considered statistically significant. Statistical analyses were performed with the R statistical software (Version 3.4.0) and SPSS 20.0 software (SPSS, Chicago, IL, USA).

Overall, 154,597 healthy participants from Beijing, China were finally enrolled in our study, including 71,892 females and 82,705 males (Supplementary Table 1). The average age of all participants was 45.5 ± 12.8 years. MGUS was diagnosed in 843 patients (0.53%, 95% CI 0.49–0.57%). The median age at presentation was 58 years (range, 25–96). The overall prevalence of MGUS was 1.11% (95% CI 1.02–1.18%) among participants aged ≥50 years, 2.57% (95% CI 2.22–2.98%) among those aged ≥70 years. In both sexes, prevalence increased with age (Fig. 1): 0.1% (<40 years), 0.36% (40–49 years), 0.78% (50–59 years), 1.28% (60–69 years), 2.19% (70–79 years), and 3.77% (≥80 years) separately (Supplementary Table 1). MGUS was found in 543 of the 82,705 men, as compared with 280 of the 71,892 women (0.66% vs 0.39%, *P* < 0.001). The prevalence among men aged ≥50 years was similar to that among women a decade older (Fig. 1).

**Fig. 1:**
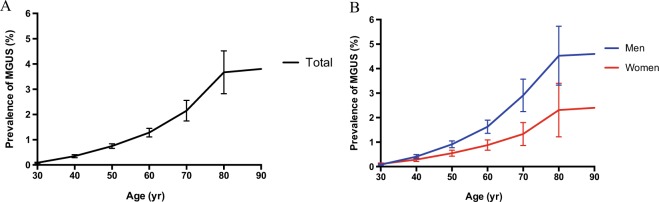
Prevalence of MGUS according to age and sex groups. **a** Prevalence of MGUS according to age groups in the Chinese population. **b** Prevalence of MGUS according to age and sex groups in the Chinese population. The I bars represent 95% confidence intervals. Years of age <30 have been collapsed to 30 years of age. Years of age >90 have been collapsed to 90 years of age.

The median concentration of serum M protein was 1.4 g/L (range, unmeasurable–27.8 g/L). The M protein level was too low to measure in 204 patients (24.79%), between 0.2 and 5 g/L for 460 (55.89%) and >15 g/L for 16 (1.9%) of the 843 MGUS patients (Supplementary Table 2). There was no significant difference in the concentration of the monoclonal protein among the age groups. Of the 602 patients who were tested for IFE, the isotype of the monoclonal immunoglobulin was IgG in 396 (65.78%), IgA in 135 (22.43%), IgM in 53 (8.80%), IgD in 2 (0.33%), light chain in 3 (0.50%), and biclonal in 13 (2.16%). The serum light-chain type was kappa in 305 (50.66%) and lambda in 255 (48.67%) patients, while 4 patients (0.66%) with biclonal M protein had both kappa and lambda light chain. Of the 440 people who were tested for FLC, 98 patients (22.27%) had an abnormal FLC ratio (<0.26 or >1.65).

The concentration of uninvolved immunoglobulins was reduced in 48 of the 313 patients (15.34%) who underwent an immunoglobulin assay. Among them, 39 (12.46%) patients had a reduction in 1 of the 2 measured uninvolved isotypes of polyclonal immunoglobulins, and 8 (2.56%) patients had a reduction in both isotypes. Only one patient with the light chain λ subtype had a reduced level of three uninvolved immunoglobulins. Urine from 165 subjects with MGUS was tested by means of immunofixation electrophoresis. A monoclonal kappa light chain (12.73%) or lambda light chain (6.67%) was found in 32 patients. Four patients (2.42%) had M protein detected in urine. One patient was found to have both M protein and kappa light chain by urine immunofixation. IgG isotype, M protein <15 g/L, and normal FLC ratio were found in 251 patients (57.05%), while the remaining 189 participants (42.95%) had 1 (137, 31.14%), 2 (51, 11.59%), or 3 (1, 0.23%) abnormal risk factors. Table 1 lists the laboratory features of MGUS patients.

**Table 1:** Characteristics of MGUS patients among people with annual medical check-ups in Beijing, China.

Characteristics	Patients, number/total number (%^a^)
Monoclonal isotype (sIFE)	602^b^
IgG	396 (65.8)
IgA	135 (22.4)
IgM	53 (8.8)
IgD	2 (0.3)
Biclonal	13 (2.2)
Light chain	3 (0.5)
Type of light chain (sIFE)	602^b^
κ	305 (50.7)
λ	293 (48.7)
κ+λ	4 (0.7)
Free light-chain ratio	440^c^
Normal (0.26–1.65)	342 (77.7)
Abnormal	98 (22.3)
M protein level and free light-chain ratio	440^c^
M protein <15 g/L and free light-chain ratio normal	340 (77.3)
M protein ≥15 g/L only	2 (0.4)
Free light-chain ratio abnormal only	90 (20.5)
Both abnormal	8 (1.8)
M isotype, M protein level, and free light-chain ratio	440^c^
IgG type, M protein <15 g/L, and free light-chain ratio normal	251 (57.1)
Any 1 abnormal	137 (31.1)
Any 2 abnormal	51 (11.6)
Any 3 abnormal	1 (0.2)
Reduced concentration of uninvolved immunoglobulins	313^d^
0	265 (84.7)
1	39 (12.5)
2	8 (2.5)
3	1 (0.3)
Type of M protein (uIFE)	165^e^
κ	21 (12.7)
λ	11 (6.7)
M protein	4 (2.4)
κ+M protein	1 (0.6)

^a^The percentage was calculated as the number of patients with MGUS divided by the number who were tested.

^b^People who had tested sIFE were studied.

^c^People who had tested free light ratio (FLC) were studied.

^d^People who had tested immunoglobulins were studied.

^e^People who had tested uIFE were studied. Among them, 37 people had positive results.

In the present study, we quantified the prevalence of MGUS among a relatively healthy population in the Beijing area. This is the largest screening study and population-based study of MGUS in the Chinese population so far. Our findings are consistent with previous studies showing that Asians have a lower incidence of MGUS compared with Whites. In the same age group (aged ≥50 years), the prevalence of our study was lower than prior MGUS prevalences in Olmsted County, USA^3^, Germany^10^, France^11^, and even the Asian country Japan^12^ (Supplementary Table 3). The considerable disparities may derive from differences in ethnicity, sampling strategies, detection techniques, environmental exposures, and lifestyles. We also noticed that the prevalence of MGUS increases with advancing age and men had a significantly higher prevalence of MGUS than women.

The concentration of monoclonal protein in our study was extremely small (median, 1.5 g/L); 24.8% of patients had unmeasurable protein concentrations, and only 1.9% had a concentration of ≥15 g/L. However, the median concentration of monoclonal protein was 5 g/L in the Olmsted County study, and 19.9% of participants had monoclonal immunoglobulin >15 g/L^3^. Because our study enrolled more young participants who may have a shorter underlying course and lower disease burden, it is reasonable to interpret these varied M protein levels by age group. The most common immunoglobulin type in MGUS identified in our study was IgG (65.8%), which was also observed in previous studies^3,11–13^.

According to Mayo Clinic model for risk of progression^14^, more than half of MGUS patients (57%) in our study had low-risk MGUS (M protein level <15 g/L, IgG type, normal FLC ratio), and 43% had intermediate risk (any one or two factors abnormal), and only one patient (0.23%) was prone to high risk of progression (three factors abnormal). However, among the Minnesotan MGUS patients, low-risk MGUS accounted for 38.8%, intermediate risk for 56.4%, and the high risk for 4.8%^1^. Recent studies demonstrated that suppression of uninvolved immunoglobulins defined by heavy/light-chain pair suppression is a risk factor for progression of MGUS^15^. Nevertheless, among patients detected for serum immunoglobulins, most (84%) had normal uninvolved immunoglobulins, in line with previous studies^3^.

In summary, our population-based study provides age- and sex-specific prevalences of MGUS in a geographically defined population that is generally representative of the Chinese healthy population. The differences in prevalence and risk factors of MGUS among various ethnic populations remain to be further explored.

## Supplementary information

Supplementary Fig. 1

Supplementary Table 1.

Supplementary Table 2.

Supplementary Table 3.
