# Supplementary material for: Prevalence of monoclonal gammopathy of undetermined significance in a large population with annual medical check-ups in China
Source: Blood Cancer J. 2020 Mar 9;10(3):34. doi: 10.1038/s41408-020-0303-8 (PMC7062721; doi:10.1038/s41408-020-0303-8)
Supplement: Supplementary file 1 — Supplementary Fig. 1 [file 41408_2020_303_MOESM1_ESM.docx]

**
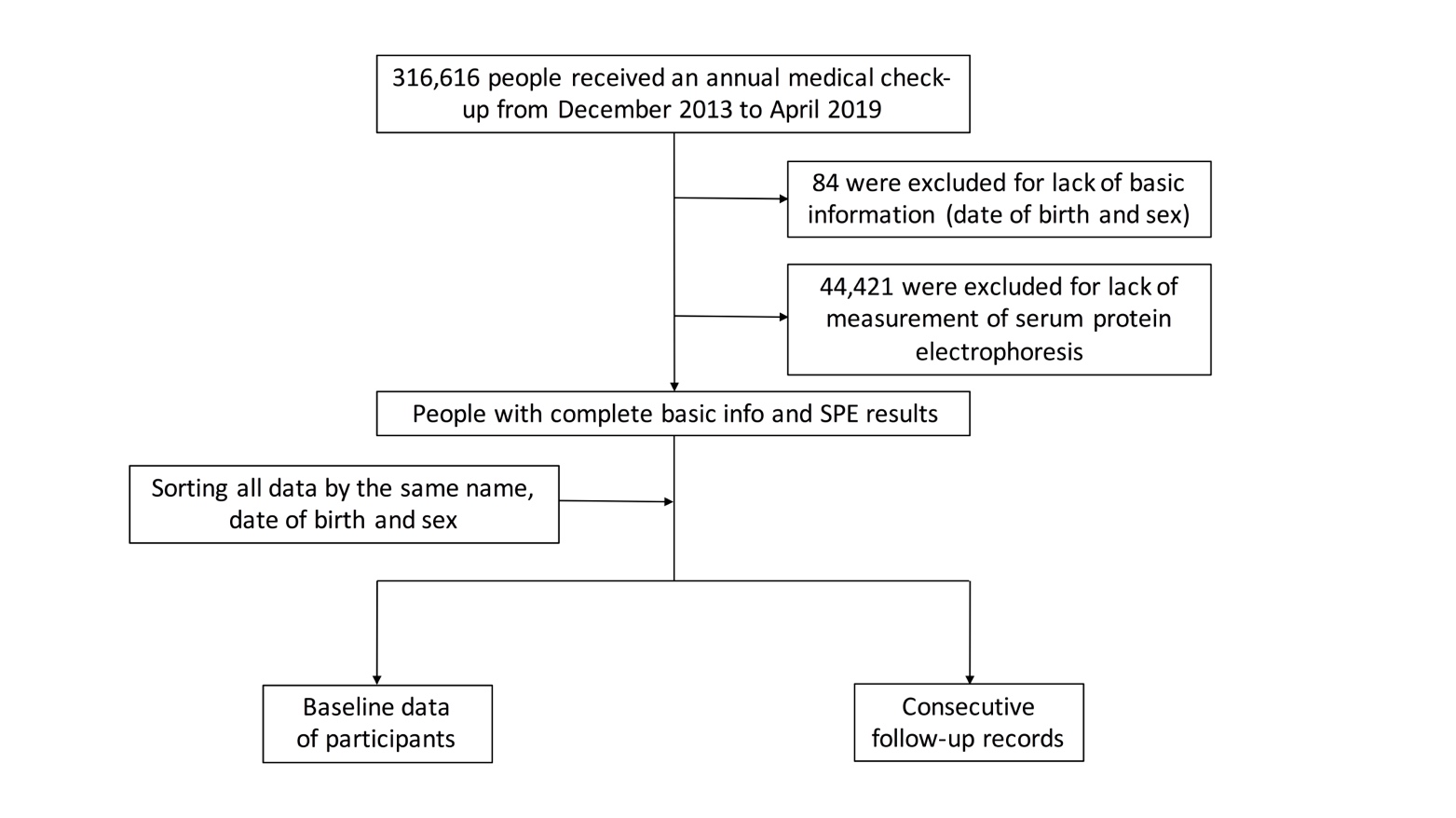
**

**Supplementary Fig. 1 Screening and sorting strategy.**

Of the 316,616 people who received an annual medical check-up in our hospital from December 2013 to April 2019, people without the measurement of serum protein electrophoresis were excluded. People were excluded if age or sex was missing in the record. We defined the same person by the same name, sex and date of birth. The information collected the first time the person came to our institute for a medical check-up was regarded as the baseline data.
