# Supplementary material for: Prevalence of monoclonal gammopathy of undetermined significance in a large population with annual medical check-ups in China
Source: Blood Cancer J. 2020 Mar 9;10(3):34. doi: 10.1038/s41408-020-0303-8 (PMC7062721; doi:10.1038/s41408-020-0303-8)
Supplement: Supplementary file 2 — Supplementary Table 1. [file 41408_2020_303_MOESM2_ESM.docx]

**Supplementary Table 1. Prevalence of MGUS according to age and sex group among people with annual medical check-ups in Beijing, China.**

| Age | Men | Women | Total |
| --- | --- | --- | --- |
| *number/total number(percent)** | | | |
| <40yr | 23/29516 (0.08) | 32/28867 (0.11) | 55/58383 (0.09) |
| 40-49yr | 87/22327 (0.39) | 50/17301 (0.29) | 137/39628 (0.35) |
| 50-59yr | 169/18664 (0.91) | 82/14966 (0.55) | 251/33629 (0.75) |
| 60-69yr | 140/8579 (1.63) | 68/7697 (0.88) | 208/16276 (1.28) |
| 70-79yr | 72/2473 (2.91) | 31/2326 (1.33) | 103/4799 (2.15) |
| ≥80yr | 52/1147 (4.53) | 17/735 (2.31) | 69/1882 (3.67) |
| <50yr | 110/51842 (0.21) | 82/46168 (0.18) | 192/98022 (0.19) |
| ≥50yr | 433/30863 (1.40) | 198/25724 (0.77) | 631/56586 (1.11) |
| ≥70yr | 124/3620 (3.43) | 48/3061 (1.57) | 172/6681 (2.57) |
| Total | 543/82705 (0.66) | 280/71892 (0.39) | 823/154597 (0.53) |
| *** The percentage was calculated as the number of patients with MGUS divided by the number who were tested. | | | |
