# Supplementary material for: Prevalence of monoclonal gammopathy of undetermined significance in a large population with annual medical check-ups in China
Source: Blood Cancer J. 2020 Mar 9;10(3):34. doi: 10.1038/s41408-020-0303-8 (PMC7062721; doi:10.1038/s41408-020-0303-8)
Supplement: Supplementary file 3 — Supplementary Table 2. [file 41408_2020_303_MOESM3_ESM.docx]

**Supplementary Table 2. Initial monoclonal (M) protein values in all MGUS patients among people with annual medical check-ups in Beijing, China.**

| M protein level (g/L) | Patients |
| --- | --- |
| *number(percent)** | |
| Unmeasurable | 204(24.79) |
| ≥0.2, <1 | 112(13.61) |
| ≥1, <5 | 348(42.28) |
| ≥5, <10 | 105(12.76) |
| ≥10, <15 | 38 (4.62) |
| ≥15, <30 | 16 (1.94) |
| *** The percentage was calculated as the number of patients with MGUS divided by the number who were tested. | |
