# Supplementary material for: Prevalence of monoclonal gammopathy of undetermined significance in a large population with annual medical check-ups in China
Source: Blood Cancer J. 2020 Mar 9;10(3):34. doi: 10.1038/s41408-020-0303-8 (PMC7062721; doi:10.1038/s41408-020-0303-8)
Supplement: Supplementary file 4 — Supplementary Table 3. [file 41408_2020_303_MOESM4_ESM.docx]

**Supplementary Table 3. Prevalence and characteristics of MGUS in previous studies.**

|  | Beijing, China  (current study) | Olmsted county,  Minnesota, USA(1, 2) | Heinz Nixdorf Recall study, German(3) | Nagasaki city, Japan(4) |
| --- | --- | --- | --- | --- |
| Population-based study | Yes  healthy adults | Yes | Yes | Yes  survivors of the atomic bomb explosion |
| Age range of study population | 25–96 years | 50 years or older | 45–75 years | 42–98 years |
| No. of person studied | 154597 | 21463 | 4708 | 52781 |
| Methods to identify M protein | Serum electrophoresis and immunofixation | Serum electrophoresis and immunofixation | Serum electrophoresis and immunofixation | Serum electrophoresis and immunofixation |
| Prevalence of MGUS (%) | 1.1  (aged 50 years or older) | 3.2  (aged 50 years or older) | 3.5  (aged 45–75 years) | 2.1  (aged 42–98 years) |
|  | | | | |
| M protein (g/L)  Median (Range) | 0.5  (unmeasurable to 27.8) | 5  (unmeasurable to 29.4) | 5.3  (unmeasurable to 22.4) | NA |
| Immunoglobulin isotype (%) |  |  |  |  |
| IgG | 65.8 | 68.9 | 58.8 | 73.6 |
| IgA | 22.4 | 10.8 | 17.0 | 17.6 |
| IgM | 8.8 | 17.2 | 17.0 | 7.5 |
| IgD | 0.3 | 0 | 0 | 0.1 |
| Biclonal | 2.2 | 3.0 | 0 | 0 |
| light-chain | 0.5 | 0 | 0 | 0 |
| Type of light chain(sIFE)  (%) |  |  |  |  |
| κ | 50.7 | 62.0 | 55.8 |  |
| λ | 48.7 | 37.9 | 43.6 |  |
| κ+ λ | 0.7 | NA | NA |  |
| Abnormal free light  chain ratio | 22.3 | 33.0 | 17.7 | NA |
| M protein level and free light-chain ratio (%) |  |  |  |  |
| M protein <15 g/L and  free light-chain ratio  normal | 77.2 | 52.8 | NA | NA |
| M protein ≥15 g/L only | 0.5 | 14.4 |  |  |
| Free light-chain ratio  abnormal only | 20.5 | 18.0 |  |  |
| Both abnormal | 1.8 | 14.8 |  |  |
| M isotype, M protein level and free light-chain ratio (%) |  |  |  |  |
| IgG type, M protein  <15 g/L and free light-  chain ratio normal | 57.1 | 38.9 | 39 | NA |
| Any 1 abnormal | 31.1 | 36.2 | 53 |  |
| Any 2 abnormal | 11.6 | 20.1 | 7 |  |
| Any 3 abnormal | 0.2 | 4.8 | 0 |  |
| Reduced concentration of uninvolved immunoglobulins (%) |  |  |  |  |
| 0 | 84.7 | 71.6 | NA | NA |
| 1 | 12.5 | 22.4 |  |  |
| 2 | 2.6 | 6.0 |  |  |
| 3 | 0.32 | 0 |  |  |
| * NA denotes not applicable | | |  |  |

**Reference**

1. Kyle RA, et al. Prevalence of monoclonal gammopathy of undetermined significance. New England Journal of Medicine. 2006;354(13):1362-9.

2. Kyle RA, et al. Long-Term Follow-up of Monoclonal Gammopathy of Undetermined Significance. New England Journal of Medicine. 2018;378(3):241.

3. Eisele L, et al. Prevalence and progression of monoclonal gammopathy of undetermined significance and light-chain MGUS in Germany. Annals of Hematology. 2012;91(2):243-8.

4. Iwanaga M, Tagawa M, Tsukasaki K, Kamihira S, Tomonaga M. Prevalence of Monoclonal Gammopathy of Undetermined Significance: Study of 52,802 Persons in Nagasaki City, Japan. Mayo Clinic Proceedings. 2007;82(12):1474-9.
